# The use of minocycline-rifampin coated central venous catheters for exchange of catheters in the setting of staphylococcus *aureus* central line associated bloodstream infections

**DOI:** 10.1186/1471-2334-14-518

**Published:** 2014-09-24

**Authors:** Anne-Marie Chaftari, Aline El Zakhem, Mohamed A Jamal, Ying Jiang, Ray Hachem, Issam Raad

**Affiliations:** Department of Infectious Diseases, Infection Control, and Employee Health, The University of Texas MD Anderson Cancer Center, Unit 1460, 1515 Holcombe Blvd., TX 77030 Houston, Texas

**Keywords:** Central venous catheters, Staphylococcus aureus, Central line associated bloodstream infections

## Abstract

**Background:**

Central venous catheters (CVC) removal and reinsertion of a new CVC in the setting of central line associated bloodstream infections (CLABSI) is not always possible in septic patients. The purpose of this study was to evaluate the outcome of patients with *Staphylococcus aureus*-CLABSI (SA-CLABSI) who had their CVCs exchanged over guidewire for minocycline/rifampin-coated (M/R)-CVC within seven days of bacteremia.

**Methods:**

Each case was matched with two control patients who had SA-CLABSI and had their CVC removed within seven days and two control patients who had their CVC retained beyond seven days. In addition, an in vitro model was developed for exchange of catheters.

**Results:**

We identified 40 patients with SA-CLABSI. Eight patients had their CVC exchanged over guidewire with M/R-CVC and were compared to 16 patients who had their CVC removed and 16 other patients who had their CVC retained. Patients who had their CVC exchanged over guidewire had a similar clinical response and relapse rates compared to patients whose CVC was removed or retained. However the rate of overall mortality was higher in patients who retained their CVC compared to those whose CVC was exchanged or removed (p = 0.034). The in vitro catheter exchange model showed that catheter exchange over guidewire using M/R-CVC completely prevented biofilm colonization compared to exchange using uncoated CVC (p < 0.0001).

**Conclusions:**

In the setting of SA-CLABSI, exchanging the CVC over guidewire with M/R-CVC could be an alternative to removing the CVC and reinserting another CVC at a different site and may be associated with a lower rate of overall mortality. Further large prospective randomized clinical trials are warranted.

**Electronic supplementary material:**

The online version of this article (doi:10.1186/1471-2334-14-518) contains supplementary material, which is available to authorized users.

## Background

Central line associated bloodstream infections (CLABSI) are a serious complication of central venous catheters (CVCs). Staphylococcus *aureus* is the second most common pathogen causing bacteremia and is associated with high morbidity and mortality in critically ill patients. Removal of the CVC is not always feasible in the setting of coagulopathy, thrombocytopenia and neutropenia. The latest Infectious Diseases Society of America (IDSA) guidelines for the treatment of CLABSI advise for exchanging the catheter for S. *aureus* bacteremia with a B-III level of recommendation [1].

We have previously demonstrated that exchanging CVC over guidewire for minocycline-rifampin coated CVC (M/R-CVC) improves the outcome of CLABSI for different pathogens [2].

In this study, we are presenting in-vivo and in-vitro data about the exchange of CVC for M/R-CVCs in the setting of S. *aureus* CLABSI.

## Methods

### Clinical data

This is a retrospective chart review of all the patients with S. *aureus* CLABSI identified at The University of Texas MD Anderson Cancer Center who had their CVC exchanged over guidewire for M/R-CVC within seven days of S. *aureus* bacteremia between January 2005 and December 2011. This retrospective study was approved by the MD Anderson Cancer Center Institutional Review Board, and a waiver of informed consent was obtained to access patients’ electronic medical records. In this matched retrospective cohort study, each case was matched with two control patients who had S. *aureus* CLABSI and had their CVC removed within seven days and two other control patients who had their CVC retained beyond seven days. Control patients were matched based on cancer type (hematological versus solid malignancy), neutropenia status and organism susceptibility. Data were collected from the institutional electronic medical records and included demographic information, clinical characteristics, laboratory data, therapy, as well as outcome. Patients were followed for 3 months from the date of bacteremia.

### In vitro exchange model experiment

In vitro exchange model was developed in our lab. Uncoated CVCs were placed in a tall glass tube (2cm×20cm) containing 75 ml of bovine calf serum for 24 h for preconditioning. After 24 h, catheters were transferred into Mueller-Hinton broth (MHB) inoculated with 1×10^5^ of MRSA, and allowed to form biofilm at 37°C for 24 h. The MRSA isolate was susceptible to minocycline and rifampin. After biofilm colonization, guidewires were inserted into the lumen of the uncoated biofilm colonized-CVCs and then the catheters were exchanged over the guidewires for fresh uncoated, M/R-, Chlorhexidine/Silver Sulfadiazine (CHX/SS)-CVCs placed in a glass tube containing 50% MHB and 50% bovine calf serum for 24 h. Six segments were cut after incubation of exchanged CVCs and sonicated in saline for 15 min. After sonication, each sample was serially diluted and spread on to Trypticase soy agar + 5% sheep blood for colony counting. The experiments were repeated three times.

#### Statistical analysis

This was a multiple-group comparison study. The chi-square or Fisher’s exact tests were used to compare categorical variables, as appropriate. If a significant result (*p* ≤ .05) was detected, pairwise comparisons between individual groups were performed to identify the significant differences. The Kruskal-Wallis test was used to compare continuous variables. If a significant result was detected, Wilcoxon rank sum tests were used for the pair-wise comparisons. The α levels of the post-hoc pairwise comparisons were adjusted using a sequential Bonferroni adjustment to control type I error. All tests except those for the pairwise comparisons were two-sided at a significance level of 0.05. The statistical analyses were performed using SAS version 9.3 (SAS Institute Inc., Cary, NC).

### Definitions

CLABSI was defined per the Centers for Disease Control and Prevention criteria [3]. Attributable mortality was defined as clinical or microbiological evidence of infection at the time of death.

Relapse is a new episode of S. *aureus* bacteremia occurring within the follow up period with an identical antibiogram to the first isolate.

## Results

The demographics and clinical characteristics of patients are outlined in Table 1.

**Table 1:** **Clinical characteristics and outcomes**

Characteristics and outcomes	CVC retained for more than 7 days since bacteremia (n = 16)	Removed within 7 days of bacteremia (n = 16)	Exchanged with antibiotic-coated catheters within 7 days of bacteremia (n = 8)	*p*-value	
	N (%)	N (%)	N (%)		
Median age (years), (range)	52 (12–74)	62 (4–83)	55 (29–67)	0.24	
Gender, male	10 (62.5%)	8 (50.0%)	2 (25.0%)	0.28	
Type of cancer				> .99	
Hematologic malignancy	6 (37.5%)	6 (37.5%)	3 (37.5%)		
Solid tumor	10 (62.5%)	10 (62.5%)	5 (62.5%)		
Body temperature				0.41	
≤ 36°C	2 (12.5%)	0 (0%)	1 (12.5%)		
36°C −38°C	6 (37.5%)	5 (31.3%)	1 (12.5%)		
≥ 38°C	8 (50.0%)	11 (68.7%)	6 (75.0%)		
MIC of vancomycin				0.77	
< 2.0	9 (56.2%)	9 (56.2%)	3 (37.5%)		
≥ 2.0	7 (43.8%)	7 (43.8%)	5 (62.5%)		
Number of lumens of catheters				0.59	
Single	7 (43.8%)	6 (37.5%)	1 (12.5%)		
Double	8 (50.0%)	9 (56.3%)	6 (75.0%)		
Triple	1 (6.3%)	1 (6.3%)	1 (12.5%)		
CVC location				0.22	
Subclavian	7 (43.8%)	6 (37.5%)	6 (75.0%)		
Jugular	2 (12.5%)	0	0		
Basilic	7 (43.8%)	10 (62.5%)	2 (25.0%)		
Defervescence within 3 days of starting antibiotics	11 (68.8%)	14 (87.5%)	6 (75.0%)	0.49	
Relapse within 3 months	2 (12.5%)	0 (0%)	1 (12.5%)	0.4	
Death within 3 months	8 (50.0%)	4 (25.0%)	0 (0%)	0.034	CVC retained vs CVC exchange: *p* = 0.02
Infection-related death within 3 months	5 (31.3%)	1 (6.3%)	0 (0%)	0.1	

In the setting of S. *aureus* CLABSI, exchange over guidewire with M/R-CVCs tended to have equivalent outcomes in terms of defervescence within three days and relapse when compared to situation where CVC was removed or retained (p = 0.49 and 0.4 respectively) (Table 1).

Overall mortality in the catheter exchange over guidewire group was similar compared to the catheter removal group and statistically better compared to the catheter retention group (p = 0.034).

**Figure 1:**
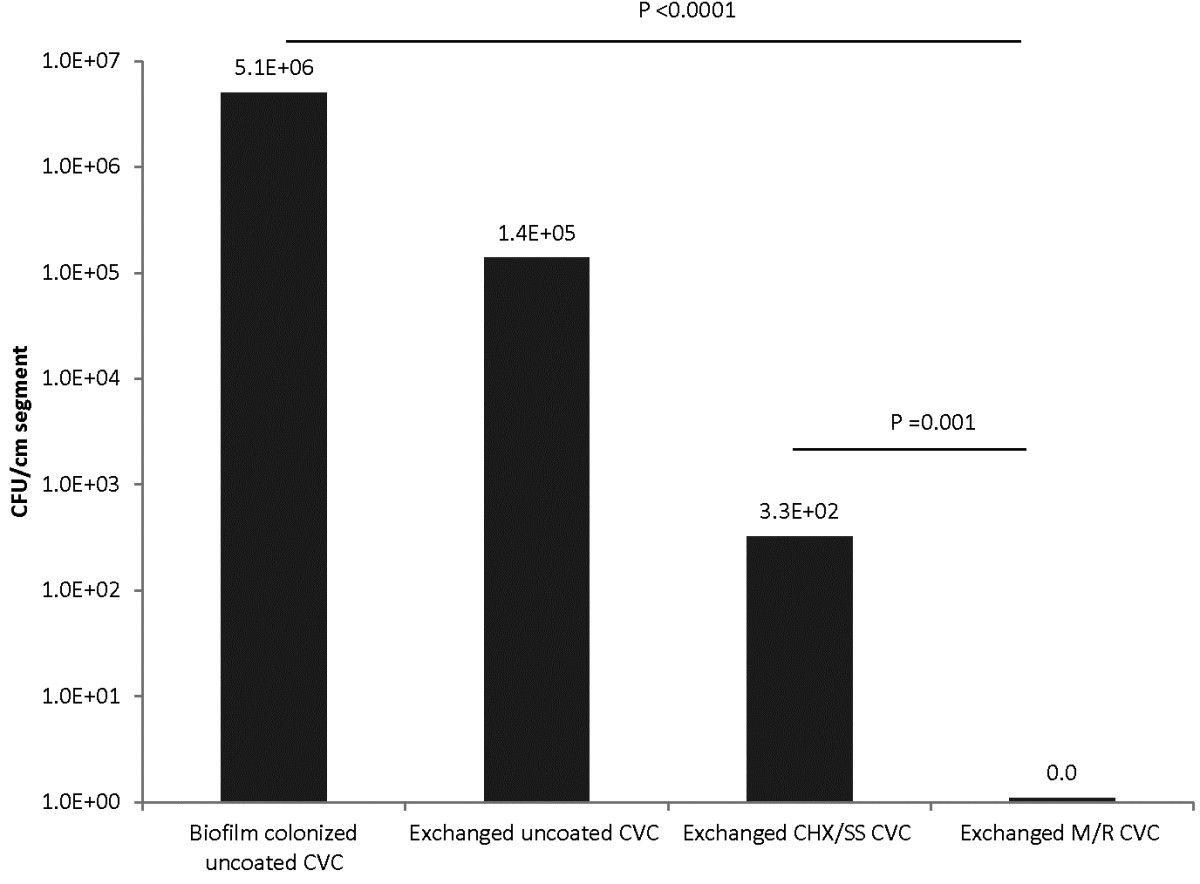
**In vitro exchange model showing efficacy of M/R, CHX/SS, and uncoated CVCs against MRSA upon exchange.** M/R, minocycline/rifampin; CHX/SS, chlorhexidine/silver sulfadiazine.

An in-vitro model of catheter exchange showed that M/R-CVC completely prevented biofilm colonization after exchange of a highly colonized CVC which was significantly better than exchange using uncoated CVC (P < 0.0001) or CHX/SS-CVCs (P = 0.001) (Figure 1).

## Discussion

S. *aureus* remains a leading cause of CLABSI in critically ill patients associated with serious complications and high mortality [4]. *Clinical Practice Guidelines for the Diagnosis and Management of Intravascular Catheter-Related Infection* caused by S. *aureus* continue to recommend early removal of the catheter and reinsertion of the new catheter at a different vascular site [1]. Failure to remove the catheter increases the risk of deep seated complications related to S. *aureus* [1].

However, in the seriously ill patients with bacteremia removal of culprit colonized CVC and reinsertion of another CVC in a different vascular site may not always be possible or achievable. Moreover, septic patients (particularly cancer and critically ill patients) either have no other vascular access or the reinsertion of a new CVC at the different site may be associated with mechanical complications such as hemopneumothorax particularly in the setting of disseminated intravascular coagulopathy (DIC) or concurrent thrombocytopenia [2]. Hence, the risk of removing the CVC and reinserting a new one might exceed the benefit of catheter removal.

The exchange of the culprit colonized CVC in the setting of CLABSI for a new uncoated CVC has been discouraged because of studies that showed transfer of organisms from the lumen of the culprit CVC to the new uncoated catheter during the exchange process [5, 6]. Our in vitro studies support this finding as they suggest that if the newly exchanged CVC is uncoated there is the risk of cross contamination (Figure 1) that would lead to the perpetuation of the infection through the newly colonized exchanged and uncoated CVC. However, in vitro and in vivo studies have shown that impregnating the internal and external surfaces of CVCs with antibiotics (M/R) prevents the surface adherence of the bacteria particularly S. *aureus*[7, 8]. These antibiotic coated CVCs were also highly effective in preventing CLABSI in high-risk patients [9, 10].

In vitro data from this current study substantiate the finding that these antibiotic impregnated catheters could be used, not only for the prevention of infection, but also for the management of infection during exchange. Our in vitro exchange model showed that M/R-CVC completely prevent the intraluminal transfer of MRSA through the guidewire during the exchange process whereas the transfer of organisms occurs when an uncoated CVC or CHX/SS-CVCs is used. The superiority of the M/R-CVC over the second generation CHX/SS is consistent with the previous findings in a large clinical trial whereby the M/R-CVC was shown to be clinically superior to the first generation CHX/SS [10].

We have recently showed that this novel approach of exchanging colonized CVC in the setting of CLABSI with a new M/R-CVC is highly useful in improving overall response rate and decreasing the risk of mechanic failure as well as disease reoccurrence [2]. However, the Chaftari et al. study had no comparative cohort in which the CVC was retained as in this current study and there was no microbiologically plausible in vitro model that supported the clinical findings.

Our clinical data show that exchange over guidewire with M/R-CVCs tended to have equivalent outcome to catheter removal in terms of response to antimicrobial therapy, relapse, mortality and infection related mortality. Concurrently, exchange over guidewire using antibiotic coated catheters as well as CVC removal showed an improved outcome in terms of mortality and possibly infection related mortality when compared to retaining the catheter (p = 0.034 and 0.1 respectively).

The clinical and in vitro data outlined above clearly highlight the safety and efficacy of exchanging a CVC over guidewire using M/R-CVCs in the setting of S. *aureus* CLABSI. This approach is particularly useful in the septic patients or the seriously ill cancer patients with either thrombocytopenia or DIC and concurrent S. *aureus* CLABSI whereby the likelihood of mechanical complications (such as hemorrhage) would be decreased by avoiding the reinsertion of another CVC in a different vascular site. In addition, the estimated comparative cost savings at our institution (the exchange vs. reinsertion procedure) is around $2250 for S. *aureus* CLABSI which has major economic implications related to the management of S. *aureus* CLABSI.

The current study is subject to several limitations. The first is the small number of patients who had undergone CVC exchange. This is due to the fact that the clinical practice guidelines recommend CVC removal for the management of S. *aureus* CLABSI. Another limitation is the observational, retrospective, nonrandomized design, although this was a matched cohort study. Patients were not on defined prospective clinical protocols, and clinical symptoms and signs were not consistently monitored. Serial blood cultures were not always available and bacteremia resolution was not always documented.

## Conclusions

In the setting of S. *aureus* CLABSI, exchanging the CVC guidewire with M/R-CVCs may be associated with a similar clinical response and relapse compared to catheter removal. However, overall mortality was lower in patients who had their CVC exchanged over a guidewire with an M/R-CVC compared to those who retained their CVC. In selected patients, this approach may serve as a safer and more cost-effective alternative to CVC removal and re-insertion of the central catheter in a different vascular site. To validate our findings, further large prospective randomized clinical trials are warranted.

## Authors’ original submitted files for images

Below are the links to the authors’ original submitted files for images. Authors’ original file for figure 1
