# Supplementary figures and images for: The use of minocycline-rifampin coated central venous catheters for exchange of catheters in the setting of staphylococcus aureus central line associated bloodstream infections
Source: BMC Infect Dis. 2014 Sep 24;14:518. doi: 10.1186/1471-2334-14-518 (PMC4261544; doi:10.1186/1471-2334-14-518)

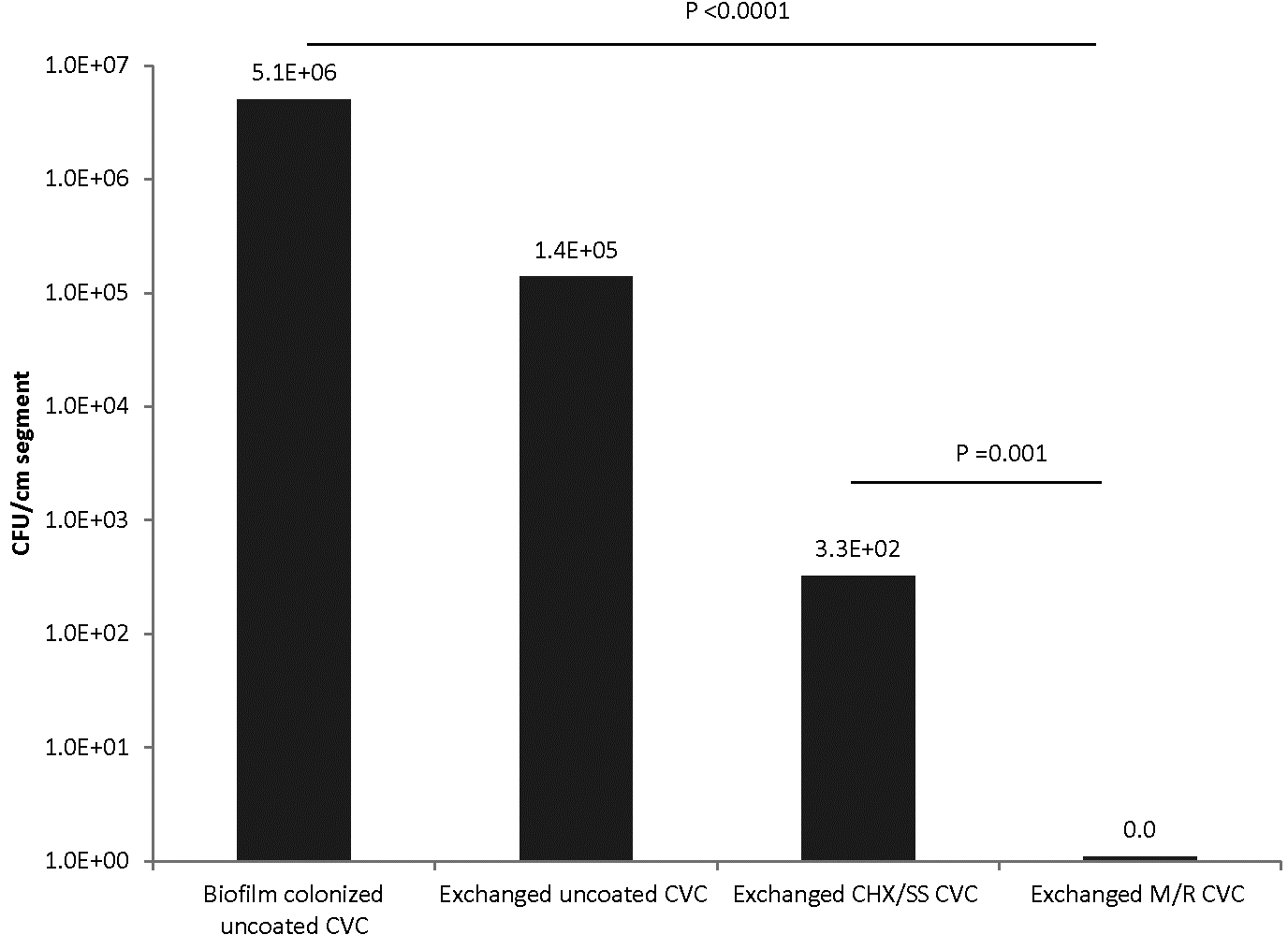

Supplement: Supplementary file 1 — Authors’ original file for figure 1 [file 12879_2014_3839_MOESM1_ESM.tiff]
